# Nodal Downstaging of Esophageal Cancer After Neoadjuvant Therapy: A Cohort Study and Meta‐Analysis

**DOI:** 10.1002/cam4.70664

**Published:** 2025-02-07

**Authors:** Feng Su, Xu Huang, Jun Yin, Hang Tang, Lijie Tan, Yaxing Shen

**Affiliations:** ^1^ Department of Thoracic Surgery, Zhongshan Hospital Fudan University Shanghai China

**Keywords:** esophageal cancer, neoadjuvant therapy, nodal downstaging, survival

## Abstract

**Background:**

In esophageal cancer, the ypN0 status after induction therapy could be categorized into two primary groups: “natural N0” (cN0/ypN0) and “down‐staged N0” (cN+/ypN0). The assessment of cN status is typically based on clinical imagination or pathological regression. However, there is no standardized method for evaluating cN/ypN status. This study aims to investigate the prognosis of patients with cN+/ypN0 using both assessment methods through a cohort study and meta‐analysis.

**Methods:**

A prospectively maintained database encompassing esophageal cancer patients undergoing induction therapy followed by radical esophagectomy was comprehensively reviewed. The prognostic significance of cN+/ypN0 across two evaluation methods was quantified. Additionally, a meta‐analysis using data from previous studies was conducted.

**Results:**

578 patients were identified from the cohort analysis, with 342 classified as ypN0 and 236 as ypN+. When evaluated with clinical imagination, patients with cN+/ypN0 had survival outcomes comparable to those with natural N0 but significantly better than those with ypN+ (*p* < 0.001). Using pathological nodal regression, cN+/ypN0 patients showed superior overall survival compared to ypN+ patients (*p* = 0.0043), although their disease‐free survival was notably inferior to that of natural N0 patients (*p* = 0.0088). A meta‐analysis of 20 previous studies confirmed the prognostic value of cN+/ypN0 status in both clinical imagination and pathological regression.

**Conclusions:**

For esophageal cancer patients receiving neoadjuvant, cN+/ypN0 status, assessed through both clinical imagination and pathological regression, serves as a significant prognostic factor. It holds precedence over ypN+ yet falls short of the natural N0. The pre‐treatment categorizations warrant recognition as a novel and pertinent staging metric.

## 1 Introduction

Preoperative therapy followed by esophagectomy is widely recommended as the standard treatment for locally advanced esophageal carcinoma [1, 2]. The 8th edition of the American Joint Committee on Cancer (AJCC) introduced the ypTNM staging system for patients who have received neoadjuvant therapy. However, compared to the pTNM used after upfront surgery, the ypTNM stage requires refinement to ensure more accurate post‐neoadjuvant staging [3]. Nodal status is a major prognostic factor in the ypStage system, particularly for patients with ypN0 status, who tend to have significantly better long‐term outcomes than those with ypN+ [4].

The ypN0 status subsequent to induction therapy could be bifurcated into two categories: “nature N0” (both clinical and postoperative N0) and “down‐staged N0” (clinical N+ and postoperative N0, cN+/ypN0). Clinical N+ status is typically assessed using imaging techniques including enhanced computed tomography (CT), endoscopic ultrasonography (EUS), and positron emission tomography (PET)/CT. Despite potential variations in nodal staging, these methods remain the most reliable preoperative tools due to their objectivity and reproducibility. Additionally, lymph nodes that are free of tumor cells but show signs of treatment response are indicative of pretreatment tumor involvement [5]. Consequently, several studies have explored the impact of down‐staged lymph nodes on the long‐term survival of esophageal cancer patients treated with induction therapy followed by radical esophagectomy. However, the findings have been inconsistent. Some studies have suggested that patients with postoperative down‐staged N0 have better prognoses than those with ypN+, but worse outcomes than those with nature N0 [6, 7]. In contrast, Davies et al. reported a significant survival advantage for patients with lymph node response [8]. Moreover, there is no consensus on which method for assessing down‐staged lymph nodes is most effective for guiding clinical decisions.

To investigate the optimal approach for evaluating down‐staged N0 and its prognostic impact on long‐term survival in patients with esophageal cancer after induction therapy, we conducted a cohort study and performed a meta‐analysis of previous studies to validate our findings.

## 2 Methods

### 2.1 Study Population

A total of 587 participants were identified from a prospectively maintained clinical database of patients with locally advanced esophageal cancer (cT2N+, or cT3‐4Nx) at the Department of Thoracic Surgery, Zhongshan Hospital, Fudan University, between January 2010 to October 2022. All patients underwent clinical staging, including enhanced CT or PET/CT and flexible upper endoscopy. After neoadjuvant therapy, patients were restaged clinically, and only those who subsequently undergone radical esophagectomy were included in the analysis. This study was conducted in accordance with the Declaration of Helsinki and approved by the Ethics Committee of Zhongshan Hospital, Fudan University. Demographic, clinical, and pathologic information was prospectively collected, encompassing details on age, gender, neoadjuvant therapy regimen, postoperative complications, tumor characteristics, lymph node status, and long‐term survival. The primary endpoint overall survival (OS) was defined as the time from randomization to death from any cause. The secondary endpoint, disease‐free survival (DFS), was defined as the time from treatment consent to disease relapse or death from any cause.

### 2.2 Stage Evaluation

Preoperative cancer staging was performed using enhanced CT, PET/CT and EUS. Lymph nodes were classified as clinical N+ if they met at least two of the following criteria: short axis > 10 mm, nonhomogeneous density, round shape, or standard uptake value > 2.5 [9, 10]. Two trained surgeons (FS and XH), blinded to the pathological status, independently reviewed the imaging data, primarily CT and PET/CT, to determine clinical nodal staging. Disagreements were resolved by a third author (HT). Pathologists from the Department of Pathology, Zhongshan Hospital, Fudan University, evaluated all surgical specimens for tumor involvement and assessed the treatment effect on the primary tumor and lymph nodes. Based lymph node status, patients were divided into three categories: patients without evidence of metastatic disease (natural N0); patients without viable cancer cells but evidence of treatment response (cN+/ypN0); and patients with positive lymph nodes (ypN+).

### 2.3 Statical Analysis

All data were analyzed with R soft version 4.4.1 (R Development Core Team, Vienna, Austria). Patients were roughly classified into two main groups: “ypN0” and “ypN+”. The “ypN0” group was further subdivided into “natural N0” and “cN+/ypN0” based on pretreatment nodal status. The clinical N was assessed with CT, PET/CT or EUS; those assessed with postoperative pathological nodal regression were termed as regression N group; ypN0 patients meeting at least one of cN+ criteria were classified as cN+/ypN0 in clinical or regression N group, while the rest were allocated to the natural N0 group. Continuous variables were presented as means ± standard deviations and compared using the t test or Wilcoxon test as appropriate. Categorical variables were recorded as frequencies with percentages, with comparisons made using the Chi‐square test or Fisher's exact test based on the data distribution. The Kaplan–Meier method and log‐rank test were employed to analyze and compare the long‐term survival between groups. Hazard ratios (HRs) with correspondent 95% confident intervals (CIs) were recorded. Statistical significance was defined as a two‐tailed *p*‐value less than 0.05.

### 2.4 Meta‐Analysis

Following the guidelines of the Preferred Reporting Items for Systematic Reviews and Meta‐Analysis (PRISMA) checklist, two electronic databases, PubMed and Web of Science, were searched for eligible trials up to October 10, 2023. The searching strategy is detailed in Table S1. The inclusion criteria were: (i) studies involving patients with locally advanced untreated esophageal cancer; (ii) patients receiving neoadjuvant chemoradiotherapy, chemotherapy, or immunotherapy followed by radical esophagectomy; (iii) patients who achieving clinical or pathological regression with cN+/ypN0 status in intervention groups; (iv) control groups consisting of natural ypN0 or ypN+ patients; (v) studies reporting long‐term outcomes. The references of included studies were also reviewed for potential inclusion. Exclusion criteria comprised case reports, small cohort studies with fewer than 5 participants and review articles. Cochrane risk of bias tools for randomized controlled trials assessed the publication biases of included articles across seven key items, including (1) random sequence generation, (2) allocation concealment, (3) blinding of participants and personnel, (4) blinding of outcome assessments, (5) incomplete outcome data, (6) selective reporting, and (7) other biases. Each item was categorized as low, unclear, or high risk of bias. Data extraction, including study author, publication year, sample size, OS and DFS (hazard ratios and 95% confidence interval) was performed by two authors (FS and XH) independently, with disagreements resolved by a third author for consensus (YXS). Heterogeneity between studies was assessed with *I* ^2^ statistic and sensitivity analyses. A two‐tailed *p*‐value < 0.05 was considered statistically significant.

## 3 Results

### 3.1 Demographic and Clinicopathological Data

The study included a total of 578 patients with an average age of 64.66 ± 7.92 years, all diagnosed with thoracic esophageal squamous cell carcinoma and treated with preoperative induction therapy. Of these, 342 patients achieved ypN0 status postoperatively, while 236 had postoperative positive nodal disease (Table 1). There were no significant differences in demographic characteristics including gender, age, postoperative complications and clinical T status (*p* > 0.05). However, the ypN+ group had a higher proportion of patients with advanced clinical N+ stage, advanced ypT stage, and evidence of lymphovascular and perineural invasion (*p* ≤ 0.001). Additionally, a greater proportion of patients in the ypN+ group received nCRT (*p* = 0.008). Detailed demographic information for ypN0 patients with various cN+ status is provided in Table S2. Among the 342 patients who achieved pathological N0, 48 showed pathological nodal regression, and 187 had positive lymph nodes in clinical assessment before therapy. Despite the high rate of false negatives in clinical evaluation, most ypN0 patients with nodal regression were classified as positive in clinical assessment (83.33%, 40/48).

**TABLE 1:** Baseline characteristics of the study participants.

Characteristics	All patients (*n* = 578)	Pathological N0 (*n* = 342)	Pathological N+ (*n* = 236)	*p*
Gender (male/female)	491 [84.9]/87 [15.1]	284 [83.0]/58 [17.0]	207 [87.7]/29 [12.3]	0.123
Age	64.66 ± 7.92	64.64 ± 7.82	64.69 ± 7.86	0.944
Neoadjuvant therapy				
nCT	275 [47.6]	147 [43.0]	128 [54.2]	0.008
nCRT	303 [52.4]	195 [57.0]	108 [45.8]	
Clinical T category				
T2	54 [9.3]	34 [9.9]	20 [8.5]	< 0.725
T3	461 [79.8]	269 [78.7]	192 [81.4]	
T4	63 [10.9]	39 [11.4]	24 [10.2]	
Clinical N category				
N0	213 [36.9]	155 [45.3]	58 [24.6]	< 0.001
N1	336 [58.1]	175 [52.1]	161 [68.2]	
N2	27 [4.7]	11 [3.2]	16 [6.8]	
N3	2 [0.3]	1 [0.3]	1 [0.4]	
Postoperative complications				
None	527 [91.2]	313 [91.5]	214 [90.7]	0.123
One or more	51 [8.8]	29 [8.5]	22 [9.3]	
ypT				
ypT0	124 [21.5]	99 [28.9]	25 [10.6]	< 0.001
ypT1	95 [16.4]	70 [20.5]	25 [10.6]	
ypT2	86 [14.9]	49 [14.3]	37 [15.7]	
ypT3	248 [42.9]	113 [33.0]	135 [57.2]	
ypT4	25 [4.3]	11 [3.2]	14 [5.9]	
Lymphovascular invasion				
Negative	458 [79.2]	301 [88.0]	157 [66.5]	< 0.001
Positive	120 [20.8]	41 [12.0]	79 [33.5]	
Perineural invasion				
Negative	440 [76.1]	277 [81.0]	163 [69.1]	0.001
Positive	138 [23.9]	65 [19.0]	73 [30.9]	

*Note:* Data are shown as mean ± SD or *n* [%].

Abbreviations: nCRT, neoadjuvant chemoradiotherapy; nCT, neoadjuvant chemotherapy; SD, standard deviation.

### 3.2 Survival Analysis

**FIGURE 1:**
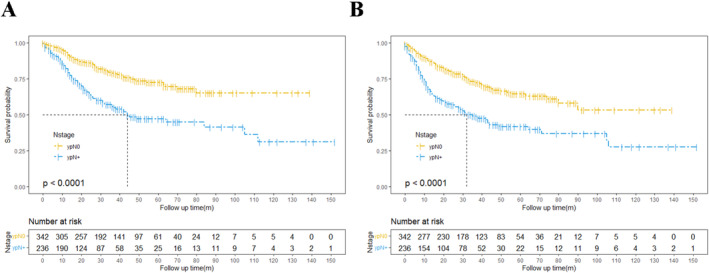
Overall survival (A) and disease‐free survival (B) curves of patients diagnosed with ypN0 and ypN+.

The median follow‐up period for all participants was 34.3 months. During this time, 173 patients died from various causes, and 206 experienced tumor recurrences. The 3‐year OS and DFS rates were 69.87% and 63.37%, while the 5‐year OS and DFS rates were 62.83% and 55.55%. The 3‐year and 5‐year OS were 78.95% and 72.59% for patient without postoperative nodal involvement, and were 55.65% and 47.23% for patients with nodal metastases, respectively. In univariate analysis, ypT stage, rather than preoperative T stage, emerged as a favorable prognostic factor for long‐term survival (*p* < 0.001, HR = 0.535 for OS and HR = 0.525 for DFS; Figure S1A–D). Patients with ypN0 had significantly better postoperative survival in both OS and DFS (OS, HR = 2.452, *p* < 0.001; DFS, HR = 2.225, *p* ≤ 0.001; Figure 1 and Table 2). Additionally, risk factors analysis identified several key prognostic factors for survival male gender (OS, HR = 1.784, *p* = 0.018; DFS, HR = 1.511, *p* = 0.046), lower ypT stage (OS, HR = 0.528, *p* < 0.001; DFS, HR = 0.522, *p* < 0.001), postoperative complications (OS, HR = 2.1873, 95% CI: 1.3020–3.6744, *p* ≤ 0.001; DFS, HR = 1.524, *p* = 0.042), lymphovascular invasion (OS, HR = 2.192, *p* < 0.001; DFS, HR = 1.756, *p* < 0.001) and perineural invasion (OS, HR = 1.839, *p* < 0.001; DFS, HR = 1.584, *p* < 0.001) were all significant for both OS and DFS, while other factors did not significantly impact long‐term outcomes. To further account for differences in clinicopathological characteristics between groups, multivariable cox regression was performed on significant risk factors. Independent risk factors for long‐term OS included ypN stage (HR = 2.075), postoperative complications (HR = 2.656) and lymphovascular invasion (HR = 1.481); for DFS, independent risk factors included ypN stage (HR = 1.916), ypT stage (HR = 0.704), and postoperative complications (HR = 1.782) (Table 2).

**TABLE 2:** Risk factors for overall survival and disease‐free survival.

	Overall survival				Disease‐free surival			
	Univariables analysis		Multivariables analysis		Univariables analysis		Multivariables analysis	
	HR (95% CI)	*p*	HR (95% CI)	*p*	HR (95% CI)	*p*	HR (95% CI)	*p*
ypN+ vs. ypN0	2.452 (1.813, 3.317)	< 0.001	2.075 (1.504, 2.865)	< 0.001	2.255 (1.712, 2.970)	< 0.001	1.916 (1.428, 2.571)	< 0.001
Age (> 60 vs. ≤ 60 years)	1.000 (0.718, 1.392)	0.994	—	—	1.094 (0.802, 1.482)	0.571	—	—
Gender (male vs. female)	1.784 (1.106, 2.877)	0.018	1.608 (0.998, 2.593)	0.512	1.511 (1.007, 2.267)	0.046	1.378 (0.918, 2.069)	0.122
Neoadjuvant therapy (nCRT vs. nCT)	0.942 (0.698, 1.271)	0.697	—	—	0.750 (0.570, 0.988)	0.041	0.908 (0.672, 1.226)	0.528
cT stage								
cT2 (ref.)	—	—	—	—	—	—	—	—
cT3	1.546 (0.926, 2.583)	0.158	—	—	1.132 (0.712, 1.799)	0.615	—	—
cT4	1.655 (0.834, 3.284)	0.165	—	—	1.060 (0.577, 1.949)	0.850	—	—
ypT stage (ypT0‐2 vs. ypT3‐4)	0.528 (0.390, 0.716)	< 0.001	0.769 (0.550, 1.075)	0.124	0.522 (0.395, 0.689)	< 0.001	0.704 (0.514, 0.964)	0.029
Postoperative complications (Yes vs. No)	2.205 (1.494, 3.256)	< 0.001	2.656 (1.785, 3.953)	< 0.001	1.524 (1.016, 2.286)	0.042	1.782 (1.169, 2.717)	0.007
Lymphovascular invasion (Yes vs. No)	2.192 (1.584, 3.034)	< 0.001	1.481 (1.023, 2.144)	0.037	1.756 (1.288, 2.394)	< 0.001	1.177 (0.825, 1.679)	0.369
Perineural invasion (Yes vs. No)	1.839 (1.324, 2.554)	< 0.001	1.387 (0.955, 2.013)	0.086	1.584 (1.164, 2.155)	< 0.001	1.197 (0.672, 1.706)	0.318

Abbreviations: CI, confidence interval; HR, hazard ratio; nCRT, neoadjuvant chemoradiotherapy; nCT, neoadjuvant chemotherapy.

**FIGURE 2:**
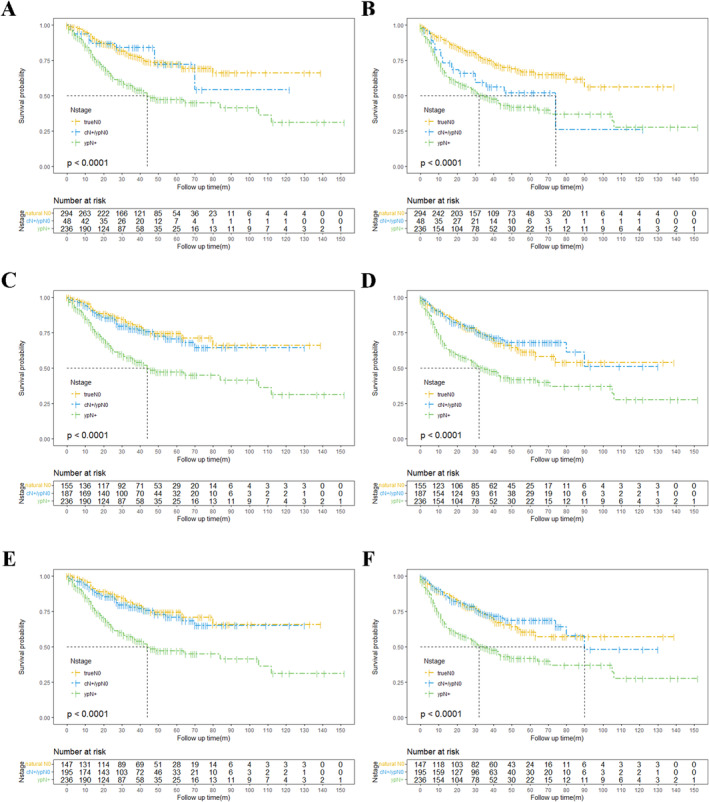
OS (A) and DFS (B) curves for patients diagnosed with natural N0, cN+/ypN0, or ypN+ disease with pathological regression; OS (C) and DFS (D) curves for patients diagnosed with natural N0, cN+/ypN0, or ypN+ disease with clinical imagination; OS (E) and DFS (F) curves for patients diagnosed with natural N0, cN+/ypN0, or ypN+ disease, considering both clinical imagination and pathological regression. DFS, disease‐free survival; OS, overall survival.

When patients were grouped by pathological nodal regression, those with natural N0 or cN+/ypN0 status had superior OS compared to ypN+ patients (natural N0, HR = 2.4047, 95% CI: 1.7541–3.2964, *p* ≤ 0.001; cN+/ypN0, HR = 2.4773, 95% CI: 1.5484–3.9634, *p* = 0.0043; Table 3). No significant differences in OS were observed between the natural N0 and cN+/ypN0 groups (HR = 0.9708, 95% CI: 0.5024–1.8756, *p* = 0.9301, Figure 2A). However, for DFS, patients in the cN+/ypN0 and ypN+ groups had significantly worse outcomes than those with natural N0 (cN+/ypN0, HR = 1.9078, 95% CI: 1.0333–3.5223, *p* = 0.0088; ypN+, HR = 2.4657, 95% CI: 1.8339–3.3151, *p* ≤ 0.001; Table 3). The DFS of patients with nodal regression was comparable to that of ypN+ patients (HR = 1.3195, 95% CI: 0.8547–2.0371, *p* = 0.2464; Figure 2B and Table 3). Subgroup analysis was conducted to account for the uneven distribution of neoadjuvant regimens across downstaging cohorts. The results confirmed that patients with ypN0 status had notably better OS compared to their ypN+ counterparts. And the survival difference between true N0 and cN+/ypN0 groups was minimal (*p* = 0.417 for nCT and *p* = 0.640 for nCRT; Figure S2A,B).

**TABLE 3:** The survival comparisons of patients in different group.

	OS		DFS	
	HR (95% CI)	*p*	HR (95% CI)	*p*
Regression N group				
ypN+ vs. natural N0	2.4047 (1.7541, 3.2964)	< 0.001	2.4657 (1.8339, 3.3151)	< 0.001
ypN+ vs. cN+/ypN0	2.4773 (1.5484, 3.9634)	0.0043	1.3195 (0.8547, 2.0371)	0.2462
cN+/ypN0 vs. natural N0	0.9708 (0.5024, 1.8756)	0.9301	1.9078 (1.0333, 3.5223)	0.0088
Clinical N group				
ypN+ vs. natural N0	2.5943 (1.8374, 3.6630)	< 0.001	2.0997 (1.5359, 2.8706)	< 0.001
ypN+ vs. cN+/ypN0	2.2660 (1.6289, 3.1523)	< 0.001	2.3222 (1.7034, 3.1658)	< 0.001
cN+/ypN0 vs. natural N0	1.1619 (0.7380, 1.8292)	0.5170	0.9067 (0.6045, 1.3598)	0.6331
Regression or clinical N group				
ypN+ vs. natural N0	2.5906 (1.8314, 3.6647)	< 0.001	2.0993 (1.5316, 2.8775)	< 0.001
ypN+ vs. cN+/ypN0	2.2276 (1.6391, 3.1649)	< 0.001	2.3111 (1.6985, 3.1448)	< 0.001
cN+/ypN0 vs. natural N0	1.1493 (0.7290, 1.8117)	0.5507	0.9157 (0.6097, 1.3752)	0.6685

In the evaluation of clinical positive nodes with CT, PET/CT or EUS, no significant difference in OS (HR = 1.1619, 95% CI: 0.7380–1.8292, *p* = 0.5170) or DFS (HR = 0.9067, 95% CI: 0.6045–1.3598, *p* = 0.6331) was found between the cN+/ypN0 and natural N0 groups. As expected, patients with natural nodal negativity had a remarkable better long‐term prognosis compared to those with postoperative nodal metastases (OS, HR = 2.5943, 95% CI: 1.8374–3.6630, *p* ≤ 0.001; DFS, HR = 2.0997 95% CI: 1.5359–2.8706, *p* ≤ 0.001). Similarly, the risk of death and relapse was significantly higher in ypN+ patients compared to the cN+/ypN0 group (OS, HR = 2.2660, 95% CI: 1.6289–3.1523, *p* ≤ 0.001; DFS, HR = 2.3222 95% CI: 1.7034–3.1658, *p* ≤ 0.001) (Figure 2C,D and Table 3). No significant difference in OS was observed between cN+/ypN0 and natural N0 groups in the subgroup analysis for neoadjuvant therapy (*p* = 0.954 for nCT and *p* = 0.129 for nCRT; Figure S2C,D).

When considering both clinical assessment and pathological regression, the clinical or regression N group showed that ypN+ status was associated with poorer outcomes compared to both natural N0 (OS, HR = 2.5906, 95% CI: 1.8314–3.6647, *p* ≤ 0.001; DFS, HR = 2.0093 95% CI: 1.5316–2.8775, *p* ≤ 0.001) and cN+/ypN0 groups (OS, HR = 2.2276, 95% CI: 1.6391–3.1649, *p* ≤ 0.001; DFS, HR = 2.3111 95% CI: 1.6985–3.1448, *p* ≤ 0.001). However, in patients who achieved ypN0 status, no significant differences in OS (HR = 1.1439, 95% CI: 0.7290–1.8117, *p* = 0.5507) or DFS (HR = 0.9157, 95% CI: 0.6097–1.3752, *p* = 0.6608) were found, regardless of the clinical N status (Figure 2E,F and Table 3). In the nCT subgroup, OS was similar between the cN+/ypN0 group and natural N0 group (*p* = 0.771, Figure S2E); however, the difference between the two groups was marginally significant in nCRT subgroup (*p* = 0.090, Figure S2).

### 3.3 Meta‐Analysis

After a thorough screening of databases and relevant citations, the meta‐analysis was conducted based on 20 studies that met the inclusion criteria [6, 7, 8, 11, 12, 13, 14, 15, 16, 17, 18, 19, 20, 21, 22, 23, 24, 25, 26, 27] (flow diagram shown as Figure S3). A total of 3309 patients were included across the studies. The majority of studies were published between 2015 and 2022, although two earlier studies were published in 2005 and 2013 [14, 27]. Eight studies focused on squamous cell carcinoma, five on adenocarcinoma, and seven included both histologic subtypes. nCRT was applied in 12 studies, and nCT was used in 6 studies, and 2 studies involved induction chemotherapy or chemoradiotherapy followed by surgery. The risk of bias assessment for these studies is available in Figure S4, with the majority of item ranked as low risk, indicating generally reliable study quality.

**FIGURE 3:**
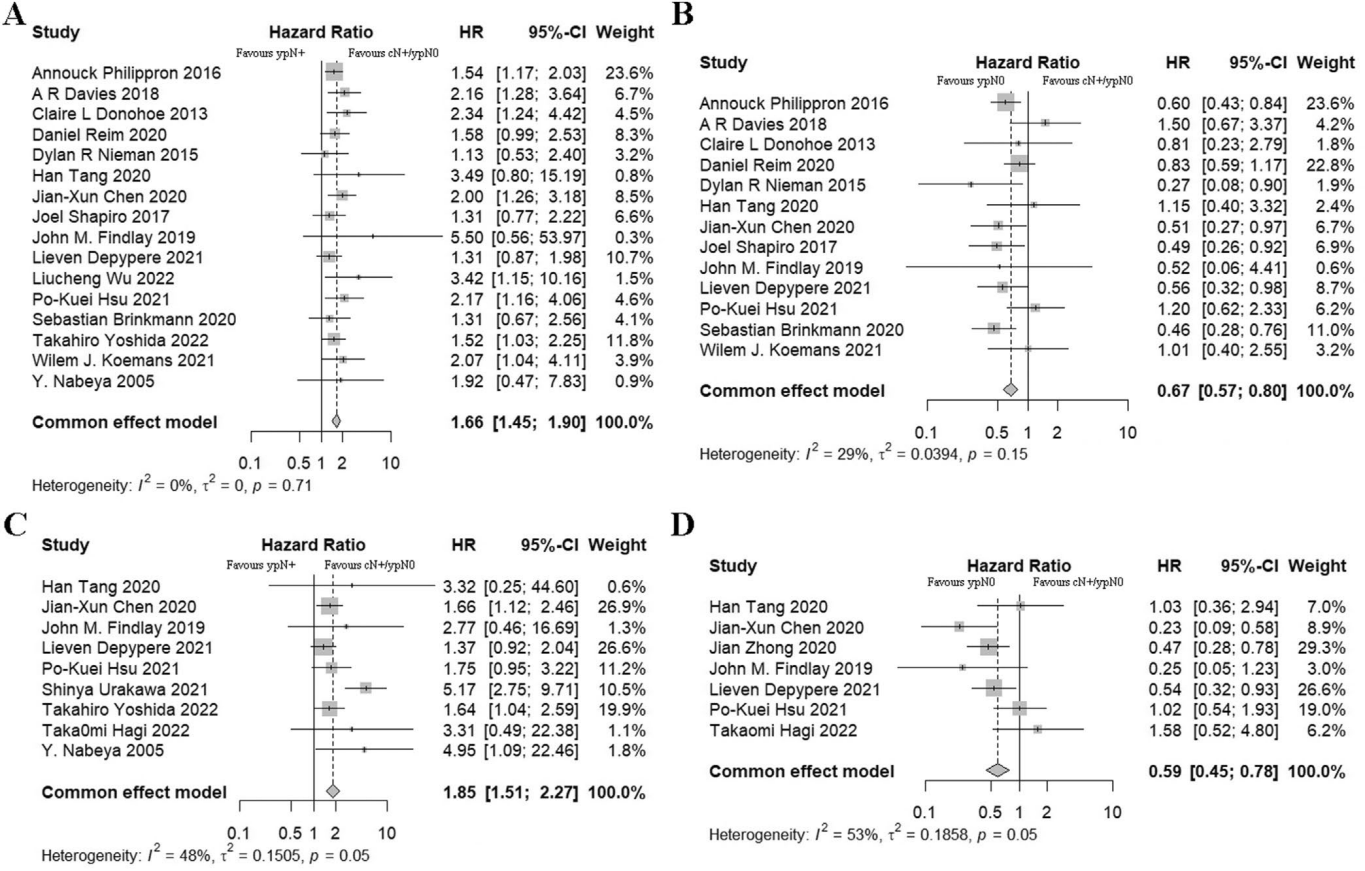
(A) Meta‐analysis of hazard ratios (HRs) for overall survival (OS) comparisons between cN+/ypN0, and ypN+ disease; (B) Meta‐analysis of HRs for OS comparisons between cN+/ypN0, and natural N0 disease; (C) Meta‐analysis of HRs for disease‐free survival (DFS) comparisons between cN+/ypN0, and ypN+ disease; (D) Meta‐analysis of HRs for DFS comparisons between cN+/ypN0, and natural N0 disease.

The included studies consistently showed that patients with cN+/ypN0 status had superior OS compared to patients with ypN+ (HR = 1.66, 95% CI: 1.45–1.90, *p* ≤ 0.001; Figure 3A), with minimal heterogeneity across the studies (*I* ^2^ = 0%, *p* = 0.71). However, the synthesis results also demonstrated that patients with natural N0 had a more favorable prognosis compared to those with cN+/ypN0, with an HR of 0.68. The heterogeneity was relatively low (95% CI: 0.57–0.80, *p* ≤ 0.001; *I* ^2^ = 29%, *p* = 0.15; Figure 3B). As for DFS, ypN+ was identified as a risk factor for long‐term survival when compared to cN+/ypN0, with moderate heterogeneity across studies (HR = 1.87, 95% CI: 1.52–2.29, *p* ≤ 0.001; *I* ^2^ = 50%, p = 0.04; Figure 3C). The summary HR also demonstrated a significantly better DFS of natural N0 compared to cN+/ypN0 (HR = 0.59, 95% CI: 0.45–0.78, *p* ≤ 0.001; Figure 3C), although there was substantial heterogeneity among the studies (*I* ^2^ = 54%, *p* = 0.04). The results of the sensitivity analysis were available in Figure S5. The synthesis effects from the sensitive analysis were consistent with those of the main analyses, further confirming the robustness of the findings.

A subgroup analysis was conducted to evaluate the importance of cN+ status as a prognosis factor. Among the 20 included studies, 9 studies scrutinized the cN+ status among ypN0 patients with diagnostic modalities including CT, enhanced CT, PET/CT or EUS [6, 12, 14, 15, 16, 21, 22, 23, 27], while the remaining 11 studies classified ypN0 patients with nodal regression [7, 8, 11, 13, 17, 18, 19, 20, 24, 25, 26]. In the subgroup evaluating clinical assessment of cN+ status, the meta‐analysis indicated that ypN+ patients had significantly inferior OS compared to cN+/ypN0 patients (HR = 2.17, 95% CI: 1.61–2.92, *p* ≤ 0.001; *I* ^2^ = 0%, *p* = 0.87; Figure S6A), while natural N0 patients had better OS than cN+/ypN0 individuals, although the difference was not statistically significant (HR = 0.66, 95% CI: 0.41–1.06, *p* = 0.08; *I* ^2^ = 0%, *p* = 0.60; Figure S6B). In studies assessing cN+ status with pathological regression, the comparisons between ypN+ and cN+/ypN0 patients demonstrated an HR of 1.56 (95% CI: 1.33–1.83, *p* ≤ 0.001; *I* ^2^ = 0%, *p* = 0.73; Figure S6C), while the HR between natural N0 and cN+/ypN0 was 0.68 (95% CI: 0.57–0.81, *p* ≤ 0.001; *I* ^2^ = 47%, *p* = 0.06; Figure S6D). Interestingly, the heterogeneity in the subgroup analysis was minimal, indicating the primary source of heterogeneity in the main analysis likely stemmed from the divergent approaches used to assess cN status. These findings collectively suggest that nodal downstaging (cN+/ypN0) is an important factor influencing patient long‐term prognosis. Additionally, the assessment method of cN+ status may affect the results. The sensitive analyses, conducted according to the different methods of assessing cN+ status, revealed relatively low levels of heterogeneity (Figure S7).

## 4 Discussion

In this retrospective study, we examined the methods used to assess cN+/ypN0 status and identified cN+/ypN0 as a distinct prognostic factor for long‐term survival in esophageal carcinoma patients treated with neoadjuvant therapy followed by curative esophagectomy. Patients classified as ypN0 with pathological regression exhibited superior OS to their ypN+ counterparts, yet their DFS was significantly worse than patients with natural N0 status. While the long‐term outcomes of ypN0 patients with cN+ on clinical imagination surpassed those of ypN+ patients. Furthermore, a comprehensive meta‐analysis incorporating data from multiple global institutions supported the finding that patients with cN+/ypN0 status may have postoperative survival outcomes superior to those of ypN+ patients, though still inferior to those with natural N0 status.

The prognostic significance of ypN status and primary tumor downstaging in esophageal cancer after induction therapy has been well established [4]. However, the effect of nodal downstaging on survival remains debated [8, 19]. Previous studies have failed to consistently demonstrate a correlation between the regression of primary tumor and nodal disease, highlighting the need for further investigation into how nodal downstaging affects survival and treatment decisions [12, 28, 29]. This discrepancy underscores the importance of accurately assessing lymph node downstaging. Notably, both suspicious lymph nodes identified through clinical imagination and pathological regression have been categorized as cN+ in prior studies, potentially contributing to conflicting findings regarding the impact of nodal downstaging. Our study conformed the prognostic strengths of cN+/ypN0, whether evaluated through clinical assessment or pathological regression.

Clinical imaging techniques, including CT, PET/CT and EUS, represent the most commonly utilized methods for preoperatively predicting lymph node metastasis in esophageal cancer. However, the accuracy is generally regarded as unreliable, with reported accuracies ranging from 59% to 80% [30, 31, 32, 33]. In contrast, the pathological assessment of lymph node regression following preoperative treatment, first introduced by Newman et al. in breast carcinoma, has become a key area of interest [34]. Several unresolved issues remain regarding pathological regression in lymph nodes. First and foremost, it is still unclear whether tumor regression also applies to nodal metastases, particularly in cases where patients are downstaged to ypN0 [35]. Additionally, the evaluation of nodal regression requires meticulous scrutiny by experienced pathologists across the entire pathological slides, constituting a laborious and time‐consuming task. Given these challenges, investigating the prognostic significance of cN+/ypN0 status and comparing it with the two methods of nodal downstaging is crucial. While neoadjuvant therapy is recommended by guidelines, the optimal neoadjuvant regimen remains underdetermined, with ongoing investigations comparing nCRT and nCT [36, 37]. Generally, it is believed that pathologic regression occurs more frequent with nCRT due to its greater aggressiveness and broader effect compared to nCT. Despite the lack of a significant difference in the superiority of cN+/ypN0 to natural N0 possibly attributable to the limited number of patients who attained ypN0 status, the results of our subgroup analysis underscored the prognostic significance of cN+/ypN0, and this finding was particularly pronounced among individuals receiving nCRT.

In this study, among the 48 ypN0 patients with pathological regression, 40 were clinically staged as having suspicious positive nodes, despite a total of 187 patients being clinically nodal positive before therapy. If we consider pathological nodal regression as the gold standard for downstage to ypN0, the specificity of clinical evaluation is only 21.4%, with a false positive rate of 78.6%. Even though clinical N‐staging is known to be relatively imprecise, these findings are significantly lower than previously reported accuracy rates [32, 33]. The proportion of ypN0 patients with nodal regression in our cohort was only 8.3%, which is considerably lower than the 20.0%–44.6% range reported in earlier studies [19, 23, 26]. Several factors may account for this discrepancy. Firstly, although our cohort was prospective maintained, most of the data was collected retrospectively over an extended period (2010–2022). Despite pathologist evaluation of all surgical specimen, some data might be incomplete. Additionally, there is currently no standardized criterion for evaluating nodal regression, primarily relying on subjective assessments of pathologists. Given the pronounced impact of nodal regression on survival outcomes, it is imperative to establish a consensus and standardized criteria for evaluating lymph node downstaging. Another plausible factor is that our cohort consists primarily of East Asian patients with esophageal squamous cell carcinoma, and the induction therapy included both nCRT and nCT. The regression patterns observed in our cohort may differ from those in European or American populations. While Zhong et al. reported an incidence of 15.8% after nCRT [7], and since pathologic downstaging is thought to occur more frequently with nCRT than nCT, the lower proportion of ypN0 patients with nodal regression in our cohort could be justifiable.

Conversely, while the DFS of ypN0 patients with pathological regression aligns with that of ypN+ patients, the long‐term outcomes of cN+/ypN0 patients often resemble those of naturally ypN0 patients in our cohort. The synthesis results from previous consistently indicate that the prognosis of cN+/ypN0 lies between that of natural N0 and ypN+ patients. The survival outcomes of ypN0 patients with clinical nodal positivity in our cohort analysis basically corroborates the results of the meta‐analysis. However, the meta‐analysis also revealed an inferior OS for ypN0 patients with nodal regression compared to those with natural N0, a distinction not observed in the cohort analysis, which may be attributed to the limited number of ypN0 patients exhibiting nodal regression observed in the cohort.

Our study has several strengths. To of our knowledge, it is the first to report and compare the prognostic impact, as well as the clinical and pathological assessment, of cN+/ypN0 in esophageal cancer patients. All patients in the study received standardized induction therapy followed by radical esophagectomy in a high‐volume center specializing in esophageal cancer. Clinical imaging, including CT and PET/CT scans, was re‐evaluated by thoracic surgeons, and lymph node staging was performed by experienced pathologists to ensure the accuracy. Additionally, we conducted a meta‐analysis of observational studies to assess the prognosis significance of cN+/ypN0. However, there are several limitations to consider. Firstly, this analysis was retrospective and based on a single‐institution, prospectively collected database, potentially introducing bias. Certain prognostic factors, such as primary tumor stage and regression, were not included in the final analysis. Furthermore, the cohort analysis was limited by a relatively small sample size of 48 individuals with nodal regression, which constrains our ability to draw definitive conclusions. Besides, the studies included in the meta‐analysis exclusively employed one of the two evaluation methods, preventing a direct comparison of their relative prognostic value. Finally, this study was unable to determine which method, clinical imagination or pathological regression, superior for evaluating cN status. Large‐scale studies with longer follow‐up durations are necessary to further validate the prognostic impact of cN+/ypN0 and the two different evaluation methods.

## 5 Conclusion

In conclusion, this cohort, along with a meta‐analysis of previous studies, provides the most comprehensive evaluation of prognosis for cN+/ypN0 esophageal cancer patients who underwent induction therapy followed by radical esophagectomy. Aligned closely with prior publications, our findings conformed the prognostic significance of cN+/ypN0, which demonstrates better outcomes than ypN+ but inferior survival compared to true N0. The evaluation of cN+/ypN0 might therefore serve as a valuable new staging parameter. The optimal assessment methods for nodal downstaging have not been determined, the evaluation of pathological regression is complicated but might retain unique survival predictive value.

## Author Contributions

**Feng Su:** conceptualization (equal), formal analysis (equal), investigation (equal), methodology (equal), resources (equal), software (equal), validation (equal), visualization (equal), writing – original draft (equal), writing – review and editing (equal). **Xu Huang:** conceptualization (equal), data curation (equal), formal analysis (equal), methodology (equal), resources (equal), software (equal), validation (equal), visualization (equal), writing – original draft (equal). **Jun Yin:** formal analysis (equal), funding acquisition (equal), investigation (equal), project administration (equal), supervision (equal), validation (equal), visualization (equal), writing – review and editing (equal). **Hang Tang:** data curation (equal), project administration (equal), resources (equal), supervision (equal). **Lijie Tan:** data curation (equal), project administration (equal), supervision (equal), validation (equal), writing – review and editing (equal). **Yaxing Shen:** data curation (equal), funding acquisition (equal), methodology (equal), project administration (equal), resources (equal), supervision (equal), validation (equal), writing – review and editing (equal).

## Ethics Statement

This study has been approved by the Ethics Committee of Zhongshan hospital, Fudan University (No. B2021‐369(2)R).

## Consent

For the retrospective nature of this study, the informed consent of patients was waived.

## Conflicts of Interest

The authors declare no conflicts of interest.

## Supporting information


**Figure S1:** (A) Overall survival (OS) curves for patients with different pretreatment T stages; (B) disease‐free survival (DFS) curves for patients with different pretreatment T stages; (C) OS curves for patients with different pathological ypT stages; and (D) DFS curves for patients with different pathological ypT stages.

**Figure S2:** OS curves for patients diagnosed with natural N0, cN+/ypN0, or ypN+ disease with pathological regression after receiving nCT (A) or nCRT (B); OS curves for patients diagnosed with natural N0, cN+/ypN0, or ypN+ disease with clinical imagination after receiving nCT (C) or nCRT (D); OS curves for patients diagnosed with natural N0, cN+/ypN0, or ypN+ disease, considering both clinical imaging and pathological regression, after receiving nCT (E) or nCRT (F). OS, overall survival; nCT, neoadjuvant chemotherapy; nCRT, neoadjuvant chemoradiotherapy.

**Figure S3:** The PRISMA flow diagram of the meta‐analysis.

**Figure S4:** Risk of bias assessment for included studies.

**Figure S5:** Sensitivity analysis for the synthesis of hazard ratios (HRs) for (A) overall survival (OS) comparisons between cN+/ypN0, and ypN+ disease, (B) OS comparisons between cN+/ypN0, and natural N0 disease, (C) disease‐free survival (DFS) comparisons between cN+/ypN0, and ypN+ disease, (D) DFS comparisons between cN+/ypN0, and natural N0 disease.

**Figure S6:** Meta‐analysis of hazard ratios (HRs) for overall survival (OS) comparisons between (A) cN+/ypN0 and ypN+ disease according to clinical imagination, (B) cN+/ypN0 and ypN0 disease according to clinical imagination, (C) cN+/ypN0 and ypN+ disease according to pathological regression, (D) cN+/ypN0 and ypN0 disease according to pathological regression.

**Figure S7:** Sensitivity analysis for the synthesis of hazard ratios (HRs) for overall survival (OS) comparisons between (A) cN+/ypN0 and ypN+ disease according to clinical imagination, (B) cN+/ypN0 and ypN0 disease according to clinical imagination, (C) cN+/ypN0 and ypN+ disease according to pathological regression, (D) cN+/ypN0 and ypN0 disease according to pathological regression.

**Data S1:** Supporting Information.

## Data Availability

All data generated or analyzed during this study are included in this published article and its Supporting Information files.
