# Supplementary figures and images for: Nodal Downstaging of Esophageal Cancer After Neoadjuvant Therapy: A Cohort Study and Meta‐Analysis
Source: Cancer Med. 2025 Feb 7;14(3):e70664. doi: 10.1002/cam4.70664 (PMC11803740; doi:10.1002/cam4.70664)

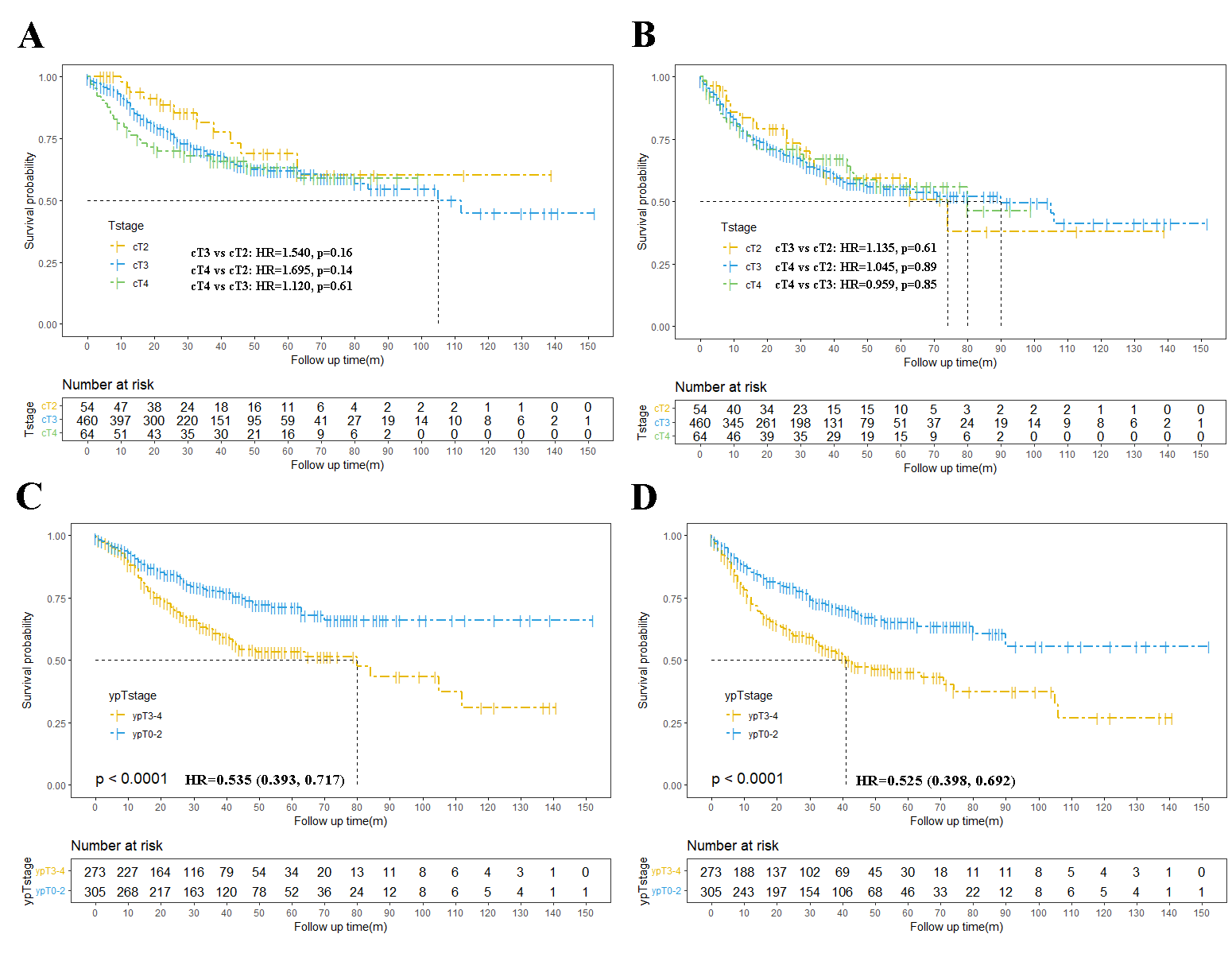

Supplement: Supplementary file 1 — Figure S1: (A) Overall survival (OS) curves for patients with different pretreatment T stages; (B) disease‐free survival (DFS) curves for patients with different pretreatment T stages; (C) OS curves for patients with different pathological ypT stages; and (D) DFS curves for patients with different pathological ypT stages. [file CAM4-14-e70664-s006.png]

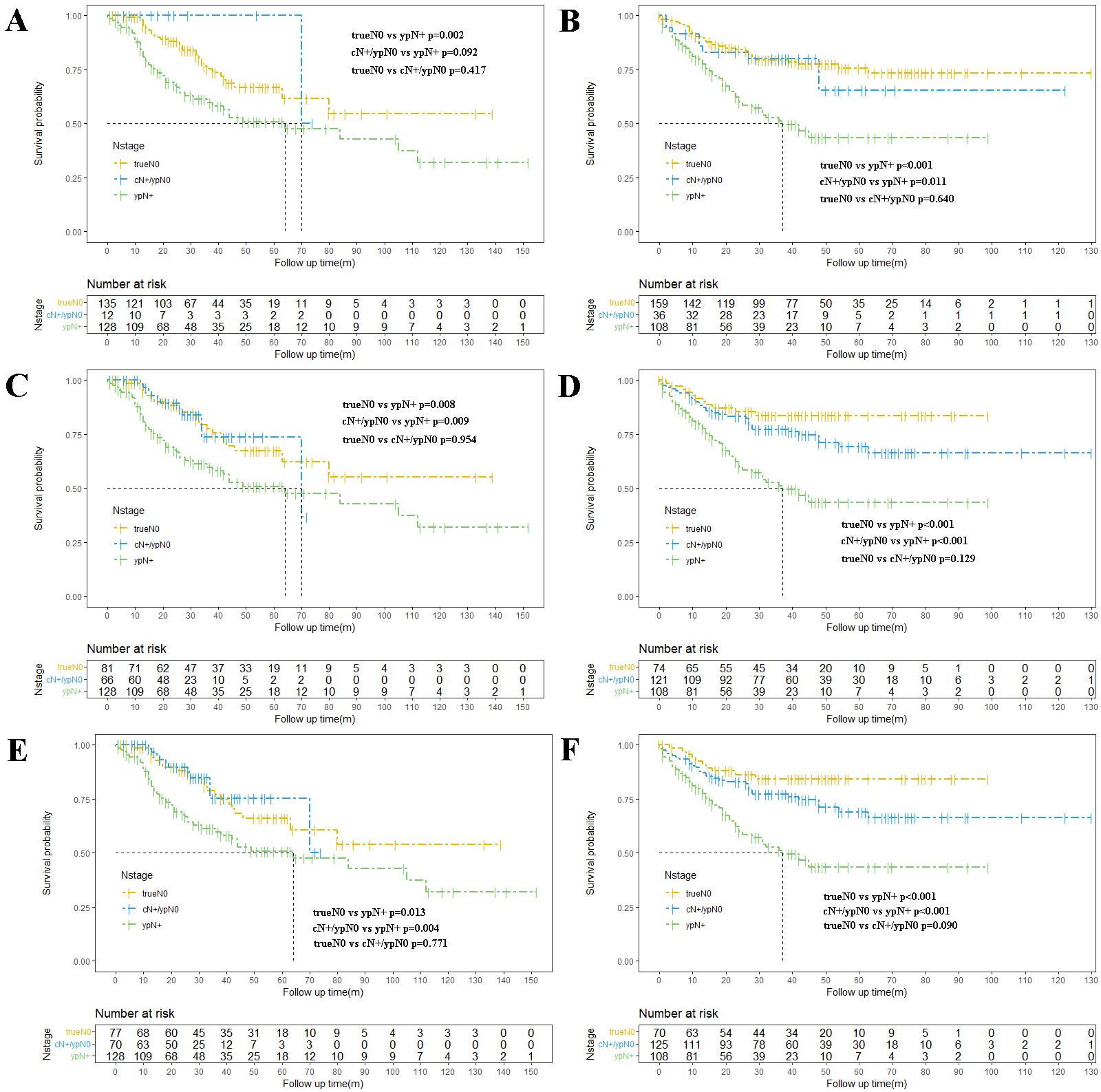

Supplement: Supplementary file 2 — Figure S2: OS curves for patients diagnosed with natural N0, cN+/ypN0, or ypN+ disease with pathological regression after receiving nCT (A) or nCRT (B); OS curves for patients diagnosed with natural N0, cN+/ypN0, or ypN+ disease with clinical imagination after receiving nCT (C) or nCRT (D); OS curves for patients diagnosed with natural N0, cN+/ypN0, or ypN+ disease, considering both clinical imaging and pathological regression, after receiving nCT (E) or nCRT (F). OS, overall survival; nCT, neoadjuvant chemotherapy; nCRT, neoadjuvant chemoradiotherapy. [file CAM4-14-e70664-s008.png]

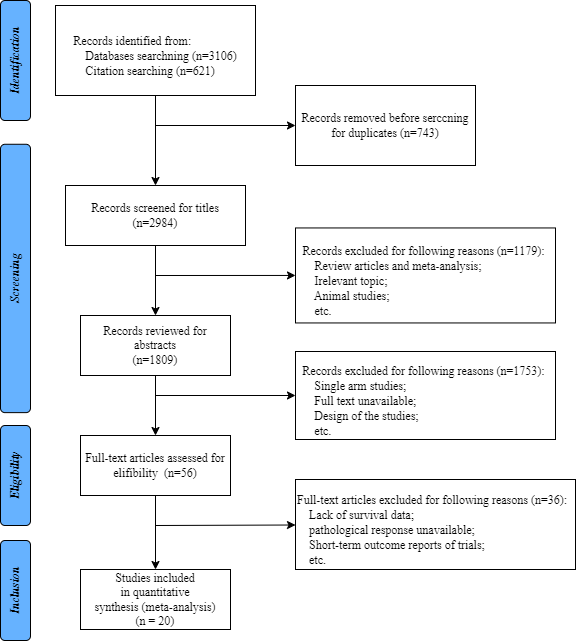

Supplement: Supplementary file 3 — Figure S3: The PRISMA flow diagram of the meta‐analysis. [file CAM4-14-e70664-s002.png]

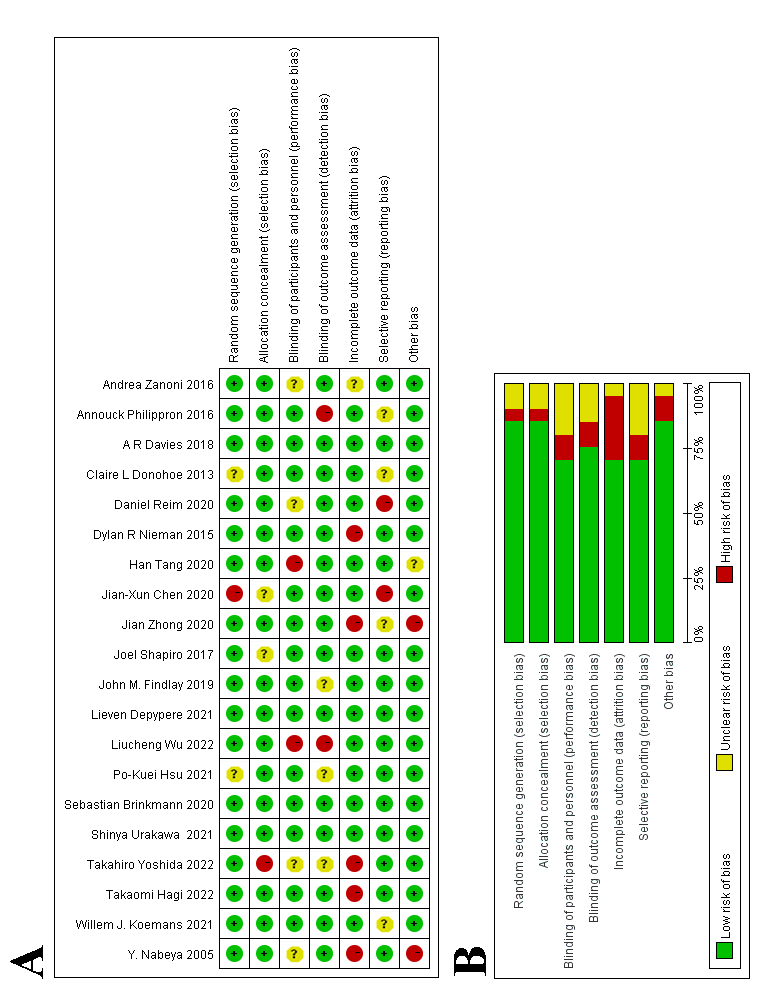

Supplement: Supplementary file 4 — Figure S4: Risk of bias assessment for included studies. [file CAM4-14-e70664-s003.png]

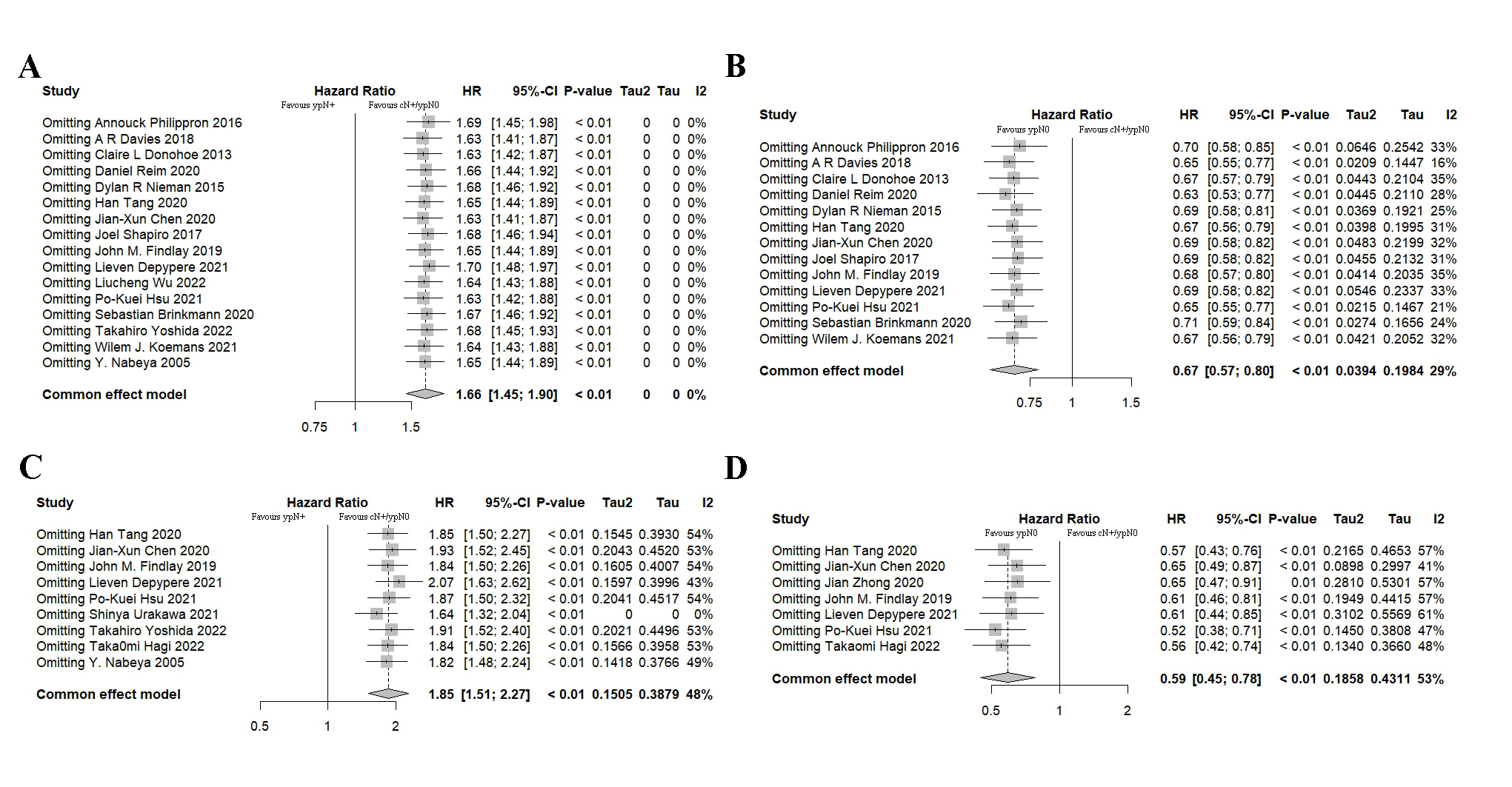

Supplement: Supplementary file 5 — Figure S5: Sensitivity analysis for the synthesis of hazard ratios (HRs) for (A) overall survival (OS) comparisons between cN+/ypN0, and ypN+ disease, (B) OS comparisons between cN+/ypN0, and natural N0 disease, (C) disease‐free survival (DFS) comparisons between cN+/ypN0, and ypN+ disease, (D) DFS comparisons between cN+/ypN0, and natural N0 disease. [file CAM4-14-e70664-s004.jpg]

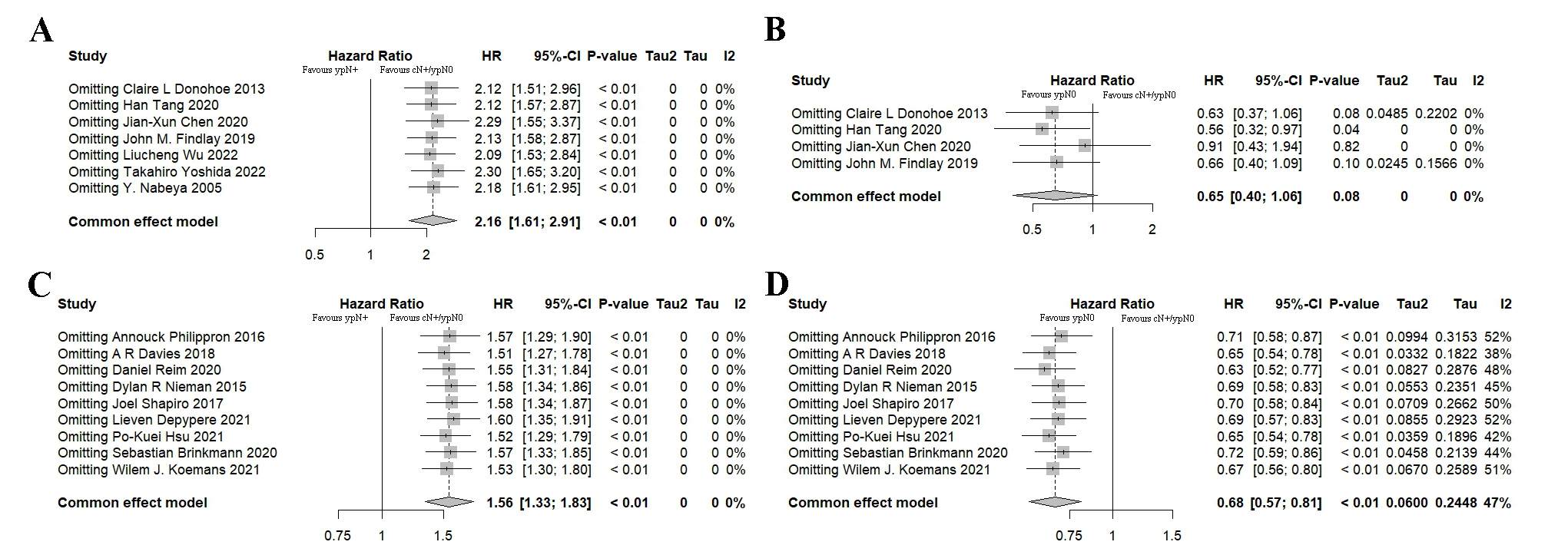

Supplement: Supplementary file 6 — Figure S6: Meta‐analysis of hazard ratios (HRs) for overall survival (OS) comparisons between (A) cN+/ypN0 and ypN+ disease according to clinical imagination, (B) cN+/ypN0 and ypN0 disease according to clinical imagination, (C) cN+/ypN0 and ypN+ disease according to pathological regression, (D) cN+/ypN0 and ypN0 disease according to pathological regression. [file CAM4-14-e70664-s005.jpg]

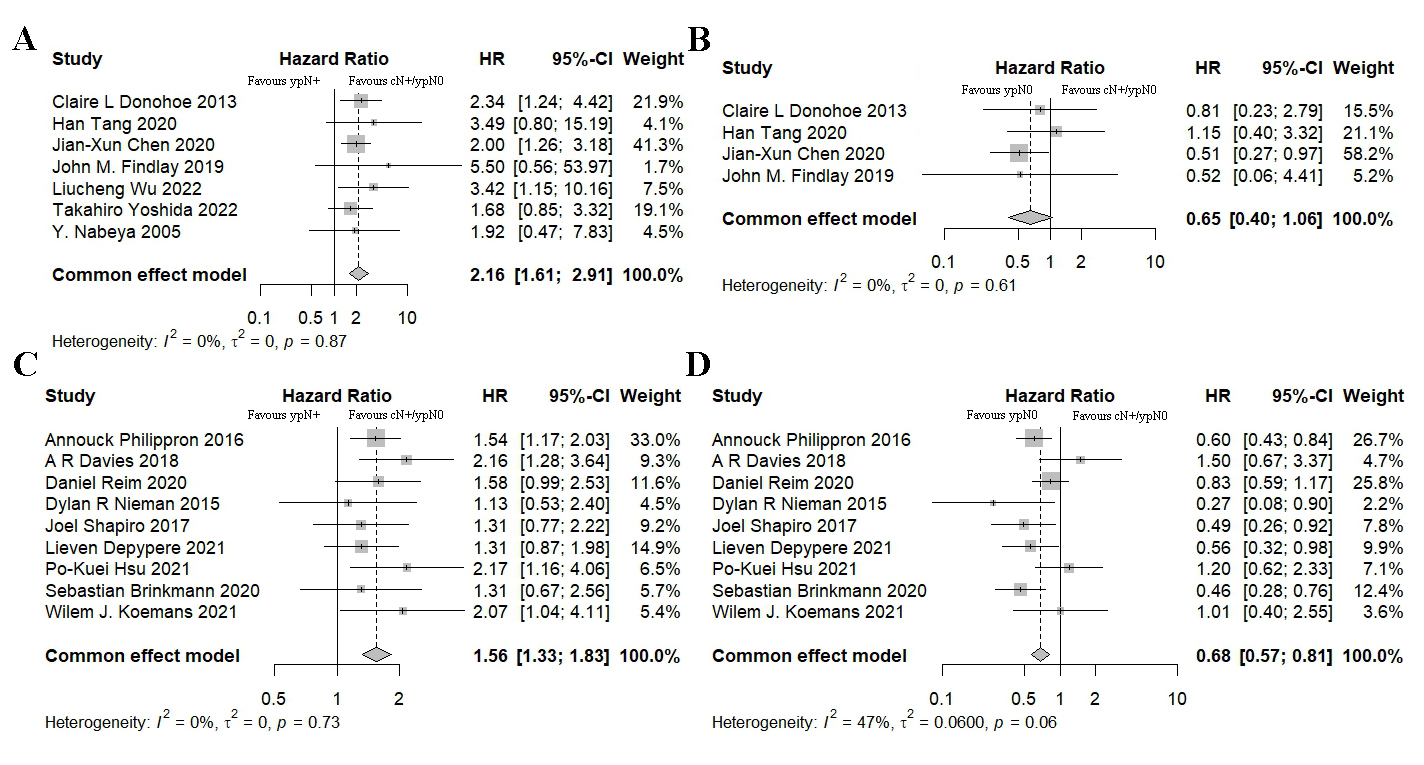

Supplement: Supplementary file 7 — Figure S7: Sensitivity analysis for the synthesis of hazard ratios (HRs) for overall survival (OS) comparisons between (A) cN+/ypN0 and ypN+ disease according to clinical imagination, (B) cN+/ypN0 and ypN0 disease according to clinical imagination, (C) cN+/ypN0 and ypN+ disease according to pathological regression, (D) cN+/ypN0 and ypN0 disease according to pathological regression. [file CAM4-14-e70664-s007.jpg]
