# Supplementary material for: Nodal Downstaging of Esophageal Cancer After Neoadjuvant Therapy: A Cohort Study and Meta‐Analysis
Source: Cancer Med. 2025 Feb 7;14(3):e70664. doi: 10.1002/cam4.70664 (PMC11803740; doi:10.1002/cam4.70664)
Supplement: Supplementary file 8 — Data S1: Supporting Information. [file CAM4-14-e70664-s001.docx]

**Supplementary Table 1. The searching strategy for the databases**

| **Database** | **Searching strategy** |
| --- | --- |
| **Pubmed** | ((((Esophageal Neoplasms [MeSH Terms]) OR (Esophageal Cancer)) OR (Esophagus Carcinoma)) OR (Esophageal Carcinoma)) AND ((((Neoadjuvant Therapy[MeSH Terms]) OR (Neoadjuvant Treatment)) OR (Neoadjuvant Chemotherapy)) OR (Neoadjuvant Radiotherapy)) AND ((((Lymph Nodes[MeSH Terms]) OR (Lymph Node)) OR (Lymph Nodal)) OR (Lymphatic Nodes)) |
| **Web of Science** | (((TS=(Esophageal Neoplasms) OR TS=(Esophageal Cancer)) OR TS=(Esophagus Carcinoma)) OR TS=(Esophageal Carcinoma)) AND (((TS=(Neoadjuvant Therapy) OR TS=(Neoadjuvant Treatment)) OR TS=(Neoadjuvant Chemotherapy)) OR TS=(Neoadjuvant Radiotherapy)) AND (((TS=(Lymph Nodes) OR TS=(Lymph Node)) OR TS=(Lymph Nodal)) OR TS=(Lymphatic Nodes)) |

**Supplementary Table 2. Baseline characteristics of the participants with ypN0**

|  | Regression N group | | | Clinical N group | | | Regression or clinical N group | | |
| --- | --- | --- | --- | --- | --- | --- | --- | --- | --- |
| Characteristics | **cN+/ypN0 (n=48)** | **natural N0 (n=294)** | **P value** | **cN+/ypN0 (n=187)** | **natural N0 (n=155)** | **P value** | **cN+/ypN0 (n=195)** | **natural N0 (n=147)** | **P value** |
| Gender (male/ female) | 44 [91.7]/ 4 [8.3] | 240 [81.6]/ 54 [18.4] | 0.086 | 157 [84.0]/ 30 [16.0] | 127 [81.9]/ 28 [18.1] | 0.620 | 165 [84.6]/ 30 [15.4] | 119 [81.0]/ 28 [19.0] | 0.372 |
| Age | 62.94±7.98 | 64.92±7.95 | 0.110 | 63.20±7.79 | 66.38±7.86 | <0.001 | 63.33±7.93 | 66.38±7.71 | <0.001 |
| Neoadjuvant therapy  nCT  nCRT | 12 [25.0]  36 [75.0] | 135 [45.9]  159 [54.1] | 0.007 | 66 [35.3]  121 [64.7] | 81 [52.3]  74 [47.7] | 0.002 | 70 [35.9]  125 [64.1] | 77 [52.4]  70 [47.6] | 0.002 |
| Clinical T category  T2  T3  T4 | 4 [8.3]  38 [79.2]  6 [12.5] | 30 [10.2]  231 [78.6]  33 [11.2] | 0.902 | 8 [4.3]  156 [83.4]  23 [12.3] | 26 [16.8]  113 [72.9]  16 [10.3] | 0.001 | 11 [5.6]  161 [82.6]  23 [11.8] | 23 [15.6]  108 [73.5]  16 [10.9] | 0.009 |
| Postoperative complications  None  One or more | 45 [93.8]  3 [6.3] | 268 [91.2]  26 [8.8] | 0.550 | 165 [88.2]  22 [11.8] | 148 [95.5]  7 [4.5] | 0.017 | 173 [88.7]  22 [11.3] | 140 [95.2]  7 [4.8] | 0.032 |
| ypT  ypT0-2  ypT3-4 | 35 [73.0]  13 [27.1] | 61 [61.3]  111 [37.8] | <0.001 | 133 [68.2]  62 [31.8] | 85 [57.8]  62 [42.1] | 0.015 | 133 [68.2]  62 [31.8] | 85 [57.8]  62 [42.1] | 0.062 |
| Lymphovascular invasion  Negative  Positive | 45 [93.8]  3 [6.3] | 256 [87.1]  38 [12.9] | 0.187 | 166 [88.8]  21 [11.2] | 135 [87.1]  20 [12.9] | 0.635 | 172 [88.2]  23 [11.8] | 129 [87.8]  18 [12.2] | 0.899 |
| Perineural invasion  Negative  Positive | 43 [89.6]  5 [10.4] | 234 [79.6]  60 [20.4] | 0.102 | 154 [82.4]  33 [17.6] | 123 [79.4]  32 [20.6] | 0.482 | 161 [82.6]  34 [17.4] | 116 [78.9]  31 [21.1] | 0.394 |

Data are shown as mean ± SD or n [%]. nCRT, neoadjuvant chemoradiotherapy; nCT, neoadjuvant chemotherapy; SD, standard deviation

**Supplementary Table 3. The basic information of studies included in the analyzed.**

| Studies | Source | Country | Histology | Study size | LN regression evaluation | Neoadjuvant strategy | Esophagectomy |
| --- | --- | --- | --- | --- | --- | --- | --- |
| Willem J. Koemans et al. 2021 (1) | Esophagus | The Netherlands | AC and SCC | 117 | Pathological regression | Carboplatin and paclitaxel + radiotherapy 1.8 Gy per fraction, 23 or 28 fractions | Minimally invasive THE woth one-feld low mediastinal LND, or MIE through Ivor-Lewis approach with two-field LND |
| Shinya Urakawa et al. 2021 (2) | Annals of Surgery | Japan | SCC | 119 | Preoperative imagination | Adriamycin, cisplatin, and 5-fluorouracil or docetaxel, cisplatin, and 5-fluorouracil | En bloc esophagectomy with two-field or three-field LND through right thoracotomy or thoracoscopy |
| Lieven Depypere et al. 2021 (3) | Annals Of Thoracic Surgery | Belgium | AC | 194 | Pathological regression | Cisplatin and 5-fluorouracil + radiotherapy between 36 to 45 Gy (1.8 Gy per fraction) | Transthoracic esophagectomy with en bloc three -field LND |
| Y. Nabeya et al. 2005 (4) | Diseases Of the Esophagus | Japan | SCC | 19 | Preoperative imagination | 5-fluorouracil and cisplatin + radiotherapy up to 46 Gy (1.8-2.0 Gy per fraction) | Esophagectomy with three-field LND |
| John M. Findlay et al. 2019 (5) | European Radiology | United Kingdom | AC and SCC | 176 | Preoperative imagination | Cisplatin/oxaliplatin and 5-fluorouracil/capecitabine or epirubicin,  cisplatin/oxaliplatin and 5-fluorouracil/capecitabine | Left thoracoabdominal, Ivor-Lewis or three-stage esophagectomy with minimum two-field LND |
| Jian Zhong et al. 2020 (6) | European Journal of Surgical Oncology | China | SCC | 193 | Pathological regression | Cisplatin based chemotherapy (vinorelbine, docetaxel, paclitaxel or fluorouracil combined with cisplatin) + radiotherapy of 40-45 Gy in 20-25 fractions | Mckeown's or Ivor Lewis or left transthoracic esophagectomy with two-field LND |
| Takahiro Yoshida et al. 2022 (7) | Anticancer Research | Japan | SCC | 51 | Preoperative imagination | Docetaxel, cisplatin and 5-fluorouracil with or without radiotherapy between 40 to 50.4 Gy (1.8-2.0 Gy per fraction) | Esophagectomy |
| Takaomi Hagi et al. 2022 (8) | Annals of Surgery | Japan | SCC | 154 | Pathological regression | Adriamycin, cisplatin, and 5-fluorouracil or docetaxel, cisplatin, and 5-fluorouracil | Subtotall esophagectomy with two-field or three-field LND |
| A R Davies et al. 2018 (9) | British Journal of Surgery | United Kingdom | AC | 186 | Pathological regression | Cisplatin and 5-fluorouracil or epirubicin, cisplatin and 5-fluorouracil or epirubicin, cisplatin and capecitabine | Esophagectomy |
| Daniel Reim et al. 2020 (10) | Journal of Pathology Clinical Research | Germany | AC | 267 | Pathological regression | Oxaliplatin, leucovorin and 5-fluorouracil or cisplatin, leucovorin and 5-fluorouracil or etoposide, adriamycin and cisplatin or modified platinum-based regimens | Ivor-Lewis abdominothoracic oesophagectomy with two-field LND |
| Dylan R Nieman et al. 2015 (11) | Annals Of Thoracic Surgery | USA | AC | 90 | Pathological regression | Chemotherapy or chemoradiotherapy | Transhiatal esophagectomy, en bloc esophagectomy through right thoracotomy or right thoracoscopic minimally invasive esophagectomy |
| Annouck Philippron et al. 2016 (12) | Seminars in Thoracic and Cardiovascular Surgery | Germany | AC and SCC | 403 | Pathological regression | Cisplatin and 5-fluorouracil + radiotherapy of 36-40 Gy (1.8 Gy per fraction) | Ivor Lewis sub-total en bloc esophagectomy with two-field LND |
| Liucheng Wu et al. 2022 (13) | Journal Of Surgical Oncology | China | AC | 125 | Preoperative imagination | 5-fluorouracil/oxaliplatin/docetaxel, capecitabine/oxaliplatin, S-1/oxaliplatin, S-1/oxaliplatin/docetaxel or 5-fluorouracil/oxaliplatin | Esophagectomy |
| Jian-Xun Chen et al. 2020 (14) | World Journal of Surgical Oncology | China | SCC | 222 | Preoperative imagination | paclitaxel and cisplatin, or cetuximab, paclitaxel and cisplatin or cisplatin and 5-fluorouracil + radiotherapy of 40-50.4 Gy (1.8-2.0 Gy per fraction) | Ivor Lewis or three-hole subtotal esophagectomy with regional LND |
| Andrea Zanoni et al. 2016 (15) | Annals Of Surgical Oncology | Italy | AC and SCC | 83 | Preoperative imagination | 5-fluorouracilcisplatin, and docetaxel + radiotherapy of 50.4 Gy | McKeown esophagectomy or proximal gastrectomy and subtotal esophagectomy |
| Sebastian Brinkmann et al. 2020 (16) | Annals Of Surgical Oncology | Germany | AC and SCC | 317 | Pathological regression | Carboplatin and paclitaxel + radiotherapy of 41.4 Gy | En bloc esophagectomy through right-sided anterolateral thoracotomy with two-field LND |
| Po-Kuei Hsu et al. 2021 (17) | Annals Of Surgical Oncology | China | SCC | 136 | Pathological regression | Cisplatin and 5-fluorouracil + radiotherapy of 41.4-50.4 Gy | McKeown tri-incisional esophagectomy |
| Han Tang et al. 2020 (18) | Journal Of Gastrointestinal Surgery | China | SCC | 110 | Preoperative imagination | Carboplatin and paclitaxel + radiotherapy of 40 Gy | Transthoracic esophagectomy with two-field LND |
| Joel Shapiro et al. 2017 (19) | Annals of Surgery | The Netherlands | AC and SCC | 180 | Pathological regression | Carboplatin and paclitaxel + radiotherapy of 41.4 Gy in 23 fractions | Transthoracic or transhiatal esophagectomy with two-field LND |
| Claire L Donohoe et al. 2013 (20) | Annals of Surgery | Ireland | AC and SCC | 167 | Preoperative imagination | Cisplatin and 5-fluorouracil + radiotherapy of 40-44 Gy | Transthoracic en-bloc esophagectomy or transhiatal esophagectomy |

LN, lymph node; AC, adenocarcinoma; SCC, squamous cell carcinoma; THE, transhiatal esophagectomy; LND, lymph node dissection

1. Koemans WJ, Larue RTHM, Kloft M, et al. Lymph node response to chemoradiotherapy in oesophageal cancer patients: relationship with radiotherapy fields. *Esophagus : Official Journal of the Japan Esophageal Society* 2021; 18(1):100-110.

2. Urakawa S, Makino T, Yamasaki M, et al. Lymph Node Response to Neoadjuvant Chemotherapy as an Independent Prognostic Factor in Metastatic Esophageal Cancer. *Annals of Surgery* 2021; 273(6):1141-1149.

3. Depypere L, De Hertogh G, Moons J, et al. Importance of Lymph Node Response After Neoadjuvant Chemoradiotherapy for Esophageal Adenocarcinoma. *The Annals of Thoracic Surgery* 2021; 112(6):1847-1854.

4. Nabeya Y, Ochiai T, Matsubara H, et al. Neoadjuvant chemoradiotherapy followed by esophagectomy for initially resectable squamous cell carcinoma of the esophagus with multiple lymph node metastasis. *Diseases of the Esophagus : Official Journal of the International Society For Diseases of the Esophagus* 2005; 18(6):388-397.

5. Findlay JM, Dickson E, Fiorani C, et al. Temporal validation of metabolic nodal response of esophageal cancer to neoadjuvant chemotherapy as an independent predictor of unresectable disease, survival, and recurrence. *European Radiology* 2019; 29(12):6717-6727.

6. Zhong J, Wang K, Fang S, et al. Prognostic impact of sterilized lymph nodes in esophageal squamous cell carcinomas after neoadjuvant chemoradiotherapy. *European Journal of Surgical Oncology : the Journal of the European Society of Surgical Oncology and the British Association of Surgical Oncology* 2021; 47(12):3074-3080.

7. Yoshida T, Nishino T, Goto M, et al. ypN0 in Patients With Definitive cN-positive Status After Preoperative Treatment Is a Prognostic Factor in Esophageal Cancer. *Anticancer Research* 2022; 42(1):195-203.

8. Hagi T, Makino T, Yamasaki M, et al. Pathological Regression of Lymph Nodes Better Predicts Long-term Survival in Esophageal Cancer Patients Undergoing Neoadjuvant Chemotherapy Followed by Surgery. *Annals of Surgery* 2022; 275(6):1121-1129.

9. Davies AR, Myoteri D, Zylstra J, et al. Lymph node regression and survival following neoadjuvant chemotherapy in oesophageal adenocarcinoma. *The British Journal of Surgery* 2018; 105(12):1639-1649.

10. Reim D, Novotny A, Friess H, et al. Significance of tumour regression in lymph node metastases of gastric and gastro-oesophageal junction adenocarcinomas. *The Journal of Pathology. Clinical Research* 2020; 6(4):263-272.

11. Nieman DR, Peyre CG, Watson TJ, et al. Neoadjuvant treatment response in negative nodes is an important prognosticator after esophagectomy. *The Annals of Thoracic Surgery* 2015; 99(1):277-283.

12. Philippron A, Bollschweiler E, Kunikata A, et al. Prognostic Relevance of Lymph Node Regression After Neoadjuvant Chemoradiation for Esophageal Cancer. *Seminars In Thoracic and Cardiovascular Surgery* 2016; 28(2):549-558.

13. Wu L, Xing Z, Huang M, et al. Nodal downstaging to ypN0 after neoadjuvant chemotherapy positively impacts on survival of cT4N+ GC/GEJ patients. *Journal of Surgical Oncology* 2022; 126(8):1403-1412.

14. Chen J-X, Lu T-Y, Fang H-Y, et al. The Impact of Pretreatment PET/CT Nodal Status on Esophageal Squamous Cell Carcinoma After Neoadjuvant Chemoradiation. *World Journal of Surgery* 2020; 44(7):2323-2331.

15. Zanoni A, Verlato G, Giacopuzzi S, et al. ypN0: Does It Matter How You Get There? Nodal Downstaging in Esophageal Cancer. *Annals of Surgical Oncology* 2016; 23(Suppl 5).

16. Brinkmann S, Noordman BJ, HÃ¶lscher AH, et al. External Validation of Pretreatment Pathological Tumor Extent in Patients with Neoadjuvant Chemoradiotherapy Plus Surgery for Esophageal Cancer. *Annals of Surgical Oncology* 2020; 27(4):1250-1258.

17. Hsu P-K, Yeh Y-C, Chien L-I, et al. Clinicopathological Significance of Pathologic Complete Lymph Node Regression After Neoadjuvant Chemoradiotherapy in Esophageal Squamous Cell Carcinoma. *Annals of Surgical Oncology* 2021; 28(4):2048-2058.

18. Tang H, Tan L, Wang H, et al. Nodal Downstaging of Esophageal Squamous Cell Carcinoma after Neoadjuvant Chemoradiotherapy: Survival Analysis if ypN0 Is Achieved. *Journal of Gastrointestinal Surgery : Official Journal of the Society For Surgery of the Alimentary Tract* 2020; 24(7):1469-1476.

19. Shapiro J, Biermann K, van Klaveren D, et al. Prognostic Value of Pretreatment Pathological Tumor Extent in Patients Treated With Neoadjuvant Chemoradiotherapy Plus Surgery for Esophageal or Junctional Cancer. *Annals of Surgery* 2017; 265(2):356-362.

20. Donohoe CL, O'Farrell NJ, Grant T, et al. Classification of pathologic response to neoadjuvant therapy in esophageal and junctional cancer: assessment of existing measures and proposal of a novel 3-point standard. *Annals of Surgery* 2013; 258(5).
